# Characterization of the Transcriptomes of Lgr5+ Hair Cell Progenitors and Lgr5- Supporting Cells in the Mouse Cochlea

**DOI:** 10.3389/fnmol.2017.00122

**Published:** 2017-04-26

**Authors:** Cheng Cheng, Luo Guo, Ling Lu, Xiaochen Xu, ShaSha Zhang, Junyan Gao, Muhammad Waqas, Chengwen Zhu, Yan Chen, Xiaoli Zhang, Chuanying Xuan, Xia Gao, Mingliang Tang, Fangyi Chen, Haibo Shi, Huawei Li, Renjie Chai

**Affiliations:** ^1^Key Laboratory for Developmental Genes and Human Disease, Ministry of Education, Institute of Life Sciences, Southeast UniversityNanjing, China; ^2^Research Institute of OtolaryngologyNanjing, China; ^3^Co-innovation Center of Neuroregeneration, Nantong UniversityNantong, China; ^4^Department of Otorhinolaryngology, Affiliated Eye and ENT Hospital, State Key Laboratory of Medical Neurobiology, Fudan UniversityShanghai, China; ^5^Key Laboratory of Hearing Medicine of National Health and Family Planning CommissionShanghai, China; ^6^Department of Otolaryngology-Head and Neck Surgery, Nanjing Drum Tower Hospital, Nanjing University Medical SchoolNanjing, China; ^7^Department of Otolaryngology-Head and Neck Surgery, Drum Tower Clinical Medical College of Nanjing Medical UniversityNanjing, China; ^8^Health Management and Policy, College of Public Health, Saint Louis University, St. LouisMO, USA; ^9^Department of Biotechnology, Federal Urdu University of Arts, Science and TechnologyGulshan-e-Iqbal, Pakistan; ^10^Department of Biomedical Engineering, Southern University of Science and TechnologyShenzhen, China; ^11^Department of Otorhinolaryngology Head and Neck Surgery, The Sixth People’s Hospital, Shanghai Jiao Tong UniversityShanghai, China; ^12^Institutes of Biomedical Sciences, Fudan UniversityShanghai, China; ^13^Shanghai Engineering Research Centre of Cochlear ImplantShanghai, China

**Keywords:** RNA-Seq, regeneration, proliferation, differentiation, sphere, gene expression

## Abstract

Cochlear supporting cells (SCs) have been shown to be a promising resource for hair cell (HC) regeneration in the neonatal mouse cochlea. Previous studies have reported that Lgr5+ SCs can regenerate HCs both *in vitro* and *in vivo* and thus are considered to be inner ear progenitor cells. Lgr5+ progenitors are able to regenerate more HCs than Lgr5- SCs, and it is important to understand the mechanism behind the proliferation and HC regeneration of these progenitors. Here, we isolated Lgr5+ progenitors and Lgr5- SCs from Lgr5-EGFP-CreERT2/Sox2-CreERT2/Rosa26-tdTomato mice via flow cytometry. As expected, we found that Lgr5+ progenitors had significantly higher proliferation and HC regeneration ability than Lgr5- SCs. Next, we performed RNA-Seq to determine the gene expression profiles of Lgr5+ progenitors and Lgr5- SCs. We analyzed the genes that were enriched and differentially expressed in Lgr5+ progenitors and Lgr5- SCs, and we found 8 cell cycle genes, 9 transcription factors, and 24 cell signaling pathway genes that were uniquely expressed in one population but not the other. Last, we made a protein–protein interaction network to further analyze the role of these differentially expressed genes. In conclusion, we present a set of genes that might regulate the proliferation and HC regeneration ability of Lgr5+ progenitors, and these might serve as potential new therapeutic targets for HC regeneration.

## Introduction

Sensorineural hearing loss is a common sensory disorder caused by the loss of hair cells (HCs). HCs are responsible for converting vibrational sound waves into the electrical impulses that are transmitted to the brain. Regeneration of damaged HCs could possibly yield a cure for sensorineural hearing loss, which still has no treatment other than prosthetic devices. Although the mature mammalian cochlea lacks the ability to regenerate HCs, new HCs are spontaneously regenerated in non-mammalian vertebrates from the resident supporting cells (SCs) that surround the HCs (Corwin and Cotanche, 1988; Balak et al., 1990; Roberson et al., 1992; Stone and Cotanche, 2007; Bermingham-McDonogh and Reh, 2011; Warchol, 2011). Previous studies have shown that some cochlear SCs in neonatal mammals are HC progenitors that possess a limited capacity to regenerate HCs (Chai et al., 2012; Shi et al., 2013; Bramhall et al., 2014; Cox et al., 2014; Wang et al., 2015). However, these SCs lose their intrinsic regenerative potential as the animal ages (Oesterle et al., 2008; Bermingham-McDonogh and Reh, 2011; Warchol, 2011; Cox et al., 2014), and as a result hearing loss tends to be permanent and incurable in mature mammals.

Supporting cells in the mouse inner ear are known to be a potential resource for HC regeneration after damage (White et al., 2006; Sinkkonen et al., 2011), and when isolated by flow cytometry neonatal SCs have the ability to proliferate and to differentiate into HCs *in vitro*. Upon HC injury, cochlear SCs also display a limited capacity to proliferate and to regenerate HCs (White et al., 2006; Sinkkonen et al., 2011). Leucine-rich repeat-containing G-protein coupled receptor 5 (Lgr5), a Wnt target gene, is a marker of endogenous stem cells in rapidly proliferating organs (Barker et al., 2007; Jaks et al., 2008). Lgr5 is widely expressed in the cochlear duct prosensory region during embryonic development, but it becomes restricted to a subset of SCs after birth (Chai et al., 2011). Recently, Lgr5+ cells in newborn mice have been shown to be a population of HC progenitors that can regenerate HCs through both direct differentiation and through mitotic regeneration (Chai et al., 2012; Shi et al., 2013; Bramhall et al., 2014; Cox et al., 2014; Hegarty et al., 2015; Wang et al., 2015; Lu et al., 2016). However, the detailed gene expression profile differences between the Lgr5+ progenitors and the Lgr5- SCs have not yet been investigated.

The current study focused on identifying the molecular mechanism behind the increased proliferation and HC regeneration ability of Lgr5+ progenitors in the neonatal mouse cochlea. We performed RNA-Seq profiling of the Lgr5+ progenitors and the Lgr5- SCs from Lgr5-EGFP-CreERT2/Sox2-CreERT2/Rosa26-tdTomato transgenic mice and identified the differentially expressed genes that might be involved in regulating proliferation, differentiation, or signaling pathways in these two cell populations. To further analyze the role of differentially expressed genes between Lgr5+ progenitors and Lgr5- SCs, we created a protein–protein interaction network using STRING (Search Tool for the Retrieval of Interacting Genes/Proteins). These datasets are expected to serve as a resource for future work in determining the detailed regulatory mechanisms behind HC regeneration.

## Materials and Methods

### Mice and Genotyping

Lgr5 EGFP-Ires-CreERT2 (stock no. 008875) (Barker et al., 2007), Sox2-CreER (stock no. 008875), and Rosa26-tdTomato (stock no. 007914) (Madisen et al., 2010) mice were obtained from the Jackson Laboratory. Transgenic mice were genotyped using genomic DNA from tail tips by adding 180 μl 50 mM NaOH, incubating at 98°C for 1 h, and adding 20 μl 1M Tris-HCl. The genotyping primers were as follows: Lgr5: (F) CTG CTC TCT GCT CCC AGT CT; wild-type (R) ATA CCC CAT CCC TTT TGA GC; mutant (R) GAA CTT CAG GGT CAG CTT GC; tdTomato: wild-type (F) AAG GGA GCT GCA GTG GAG T; (R) CCG AAA ATC TGT GGG AAG TC; mutant (F) GGC ATT AAA GCA GCG TAT C; (R) CTG TTC CTG TAC GGC ATG G. Sox2-CreER mutant (F) GCG GTC TGG CAG TAA AAA CTA TC; Sox2-CreER mutant (R) GTG AAA CAG CAT TGC TGT CAC TT; Sox2-CreER wild-type (F) CTA GGC CAC AGA ATT GAA AGA TCT; Sox2-CreER wild-type (R) GTA GGT GGA AAT TCT AGC ATC ATC C. Tamoxifen (Sigma, diluted in corn oil) was injected i.p. at post-natal day (P) 2 at 0.1 mg/g bodyweight to induce the Cre recombinase activity, and the cochleae were harvested at P4. All animal procedures were performed according to protocols approved by the Animal Care and Use Committee of Southeast University and were consistent with the National Institutes of Health Guide for the Care and Use of Laboratory Animals. All efforts were made to minimize the number of animals used and to prevent their suffering.

### Immunostaining

The dissected cochleae were fixed in 4% paraformaldehyde for 1 h at room temperature, washed three times with 1 × PBST (0.1% Triton X-100 in PBS), and incubated for 1 h at room temperature in blocking medium (1% Triton X-100, 1% BSA, 10% heat-inactivated donkey serum, and 0.02% sodium azide in PBS at pH 7.2). The primary antibody was diluted in PBT1 (10% Triton X-100, 1% BSA, 5% heat-inactivated goat serum, and 0.02% sodium azide in PBS at pH 7.2) and incubated with the samples overnight at 4°C. The samples were washed three times with 1 × PBST, and the secondary antibody [diluted in PBT2 (0.1% Triton X-100 and 1% BSA in PBS at pH 7.2)] was added for 1 h at room temperature. The samples were washed again three times with 1 × PBST and then mounted on slides in DAKO. Cells were imaged with an LSM 700 confocal microscope. The antibodies used in this paper were anti-myosin7a (Proteus Bioscience, #25-6790, 1:1000 dilution), anti-sox2 (Santa Cruz, #sc-17320, 1:500 dilution), anti-myosin6 (Proteus Biosciences, #25-6791, 1:500 dilution), anti-parvalbumin (Sigma, #P3088), FM1-43 dye (Invitrogen, # F35355), anti-espin1 (Transduction Labs, 1:200 dilution), Alexa Fluor^®^ 647 donkey anti-goat IgG (H+L; Invitrogen, A-21447, 1:500 dilution), and Alexa Fluor^®^ 555 donkey anti-rabbit IgG (H+L; Invitrogen, A-31572, 1:500 dilution). Cell proliferation was measured with the Click-it EdU imaging kit (Invitrogen).

### Flow Cytometry

We used the Lgr5-EGFP-CreERT2/Sox2-CreERT2/Rosa26-tdTomato transgenic mice to isolate the Lgr5+ HC progenitors and the Lgr5- SCs. Tamoxifen (Sigma, diluted in corn oil) was injected at P2, and the mice were sacrificed at P4. The cochleae were dissected in cold 1 × HBSS (Gibco) and transferred to 50 μl 1 × PBS in 1.5 ml Eppendorf tubes, and the tissues were incubated in 50 μl 0.25% trypsin-EDTA (Invitrogen; #25200-056) for 8 min at 37°C. The digestion was stopped by the addition of 50 μl trypsin inhibitor (Worthington Biochem, #LS003570), and 200 μl blunt tips (Eppendorf, #22491245) were used to triturate the tissue into single cell suspensions. The cells were filtered through a 40 μl strainer (BD Biosciences, 21008-949) to eliminate clumps, and the EGFP+ cells were sorted on a BD FACS Aria III (BD Biosciences).

### Quantitative Real-Time PCR

After FACS sorting, the Cells-to-cDNA II kit (Ambion, AM 1722) was used to extract total RNA from the collected cells and to reverse transcribe it into cDNA using oligo(dT) primers. The SYBR Green PCR Master Mix (Roche) was used on a BIO-RAD C1000 Touch thermal cycler (BIO-RAD). Expression levels of Lgr5, Sox2, and Brn3.1 were normalized to the β-actin in the same samples. The primers were as follows:

Lgr5 (F) 5′-TCT TCA CCT CCT ACC TGG ACC T-3′; Lgr5 (R) 5′-GGC GTA GTC TGC TAT GTG GTG T-3′; Sox2 (F) 5′-ATG AAC GGC TGG AGC AAC GGC A-3′; Sox2 (R) 5′-TCA CAT GTG CGA CAG GGG CAG T-3′; Brn3.1 (F) 5′-ACC CAA ATT CTC CAG CCT ACA C-3′; Brn3.1 (R) 5′-GGC GAG ATG TGC TCA AGT AAG T-3′; E2f1(F) 5′-TCA CTA AAT CTG ACC ACC AAA CG-3′; E2f1(R) 5′-TTG GAC TTC TTG GCA ATG AGC-3′; Rad51(F) 5′-GTC CAC AGC CTA TTT CAC G-3′; Rad51(R) 5′-GCA TAA GCA ACA GCC TCC-3′; Aurka(F) 5′-CTT TCC CTG ACT TTG TGA C-3′; Aurka(R) 5′-CAG TGT TTC AGT CCC TTT C-3′; Ccnf(F) 5′-AGC ACA AAG CCT TGC CAC CAT C-3′; Ccnf(R) 5′-AAG CCA GGT GCG TGT CCT TGT C-3′; Cdkn3(F) 5′-GGA CTC CTG ACA TAG CCA GC-3′; Cdkn3(R)5′-CTG TAT TGC CCC GGA TCC TC-3′; Trp63(F) 5′-TGG CAT TAG CCA TAG GTA GGC ACA-3′; Trp63(R)5′-TCA CCA CCA AGT GAA GGA ATC CCA-3′; Barhl1(F) 5′-GGT ACC AGA ACC GCA GGA-3′; Barhl1(R) 5′-TGG AGC GCC GAG TAA TTG-3′; Lbx2(F) 5′-GAG CGG CGA TTC GTC TTC-3′; Lbx2(R) 5′-TGT CTG GCA GTG CTG GGT A-3′; Nhlh1(F) 5′-GAC TCC AGT TCT GGA CTA AGT AAG-3′; Nhlh1(R) 5′-GGA CCA CTC CTG GAT CCC CGG ATC-3′; Egr4(F) 5′-TTG AGC TGG GCT TTG AAC A-3′; Egr4(R) 5′-AGA TGC CCG ACA TGA GGT T-3′; Foxd3(F) 5′-CCC ATC ACG GAC AGC CTC AG-3′; Foxd3(R) 5′-TAG GCT GTT CTT GGG CTT GC-3′; Pou3f1(F) 5′-GCG AGC ACT CGG ACG AGG AT-3′; Pou3f1(R) 5′-CGC AGA CGG CTT GGG ACA CT-3′; Dlx2(F)5′-GCC TCA CCC AAA CTC AGG T-3′; Dlx2(R)5′-AGG CAC AAG GAG GAG AAG C-3′; Lta(F)5′-ATG GCA TCC TGA AAC CTG-3′; Lta(R)5′-CCT GGG AGT AGA CAA AGT AGA G-3′; Tshz2(F) 5′-TCC AGT CCC AAC TCA AGC-3′; Tshz2(R) 5′-CCA GGT CAG AAA GCA GGT-3′; Disp2(F)5′-CCT TTG AAC GCT TTG ACG-3′; Disp2(R)5′-GCC AGG TTG CCA TGA GTA-3′; Erbb4(F) 5′-GCC CTC AAC CAG TTT CGT-3′; Erbb4(R) 5′-AGC AGC CTC CAG CAC ATT-3′; Hhip(F) 5′-CCC ATC GGC TCT TCA TTC TA-3′; Hhip(R)5′-CCT TTC GTC TCC TCC CTT TA-3′; Ihh(F) 5′-TGA CAG AGA TGG CCA GTG AG-3′; Ihh(R)5′-AGA GCT CAC CCC CAA CTA CA-3′; Wnt6(F) 5′-GCG GAG ACG ATG TGG ACT TC-3′; Wnt6(R) 5′-ATG CAC GGA TAT CTC CAC GG-3′; Dll1(F) 5′-TCA GAT AAC CCT GAC GGA GGC-3′; Dll1(R) 5′-AGG TAA GAG TTG CCG AGG TCC-3′; Dll3(F) 5′-GAT GCC TTT TAC CTG GGC CTG-3′; Dll3(R) 5′-ATC GAA GCC CGT AGA ATC CC-3′; Dll4(F)5′-CAG TTG CCC TTC AAT TTC ACC T-3′; Dll4(R)5′-AGC CTT GGA TGA TGA TTT GGC-3′; Figf(F) 5′-CCC ATC GCT CCA CCA GAT TT-3′; Figf(R) 5′-CGC ATG TCT CTC TAG GGC TG-3′; Dkk1(F)5′-TAT GAG GGC GGG AAC AAG-3′; Dkk1(R)5′-GAT GAT CGG AGG CAG ACG-3′; Wnt4(F) 5′-AGG AGT GCC AAT ACC AGT TCC-3′; Wnt4(R)5′-TGT GAG AAG GCT ACG CCA TA-3′; β-actin (F) 5′-ACG GCC AGG TCA TCA CTA TTG-3′; β-actin (R) 5′-AGG GGC CGG ACT CAT CGT A-3′.

### Sphere Assay and Differentiation Assay

The flow-sorted cells were diluted to 2 cells/μl in DMEM/F12 medium with 1% N2 (Invitrogen, 17502-048), 2% B27 (Invitrogen, 17504-044), EGF (20 ng/ml; Sigma, E9644), IGF (50 ng/ml, Sigma, I8779), heparan sulfate (20 ng/ml, Sigma, H4777), β-FGF (10 ng/ml, Sigma, F0291), and 0.1% ampicillin (Sigma, A9518-5G) and cultured in Costar ultra-low attachment dishes (Costar, 3599) for 5 days and then passaged to the next generation.

For the differentiation assay, we differentiated both flow-sorted cells and spheres. In the cell-differentiation assay, the flow-sorted Lgr5+ progenitors and Lgr5- SCs were cultured to a density of 50 cells/μl on laminin-coated plates using DMEM/F12 medium with 1% N2 (Invitrogen, 17502-048), 2% B27 (Invitrogen, 17504-044), EGF (20 ng/ml; Sigma, E9644), IGF (50 ng/ml, Sigma, I8779), heparan sulfate (20 ng/ml, Sigma, H4777), β-FGF (10 ng/ml, Sigma, F0291), and 0.1% ampicillin (Sigma, A9518) for 10 days. EdU (10 μM, Invitrogen, C10340) was added during the culture in order to label the dividing cells. In the sphere-differentiation assay, the spheres were plated on laminin-coated four-well dishes and cultured in DMEM/F12 medium with 1% N2 (Invitrogen, 17502-048), 2% B27 (Invitrogen, 17504-044), and 0.1% ampicillin (Sigma, A9518) for 10 days.

### RNA Extraction for RNA-Seq Analysis

Approximately 5,000 Lgr5+ HC progenitors and 5,000 Lgr5- SCs were isolated by FACS and split into three fractions for separate replicates. RNA-Seq libraries of FACS-purified cells were generated using the SMART-Seq v4 Ultra Low Input RNA Kit for Sequencing and the Illumina mRNA-Seq Sample Prep Kit. FACS-purified cells were suspended in 10 × lysis buffer. First strand and second strand cDNA synthesis, adaptor ligation, and PCR amplification were performed using the Illumina mRNA-Seq Sample Prep Kit. SPRI beads (Ampure XP, Beckman) were used in each purification step after RNA fragmentation for size selection. All libraries were analyzed for quality and concentration using an Agilent Bioanalyzer. Sequencing was performed using the Illumina HiSeq2500 150-bp Paired-End Platform, and FASTQ files of paired-end read files were generated.

### Data Analysis

RNA-Seq reads in the FASTQ files were trimmed using Trimmomatic. Clean reads were mapped to the mouse reference genome (mm9) using TopHat (Trapnell and Schatz, 2009) followed by transcript assembly and differential gene expression analysis using Cufflinks. Genes and transcripts were annotated using the RefGene database (NCBI). Genes with *p*-values < 0.05 were marked as significant. To assess the extent of functional enrichment, we performed gene ontology (GO) analysis with the functional annotation tool DAVID 6.7 (Huang da et al., 2009), which determines whether biological processes are enriched within a list of genes.

### Statistical Analysis

All of the data are shown as the mean ± SD, and statistical analyses were conducted using GraphPad Prism6 software. For all experiments, n represents the number of replicates, and at least three individual experiments were conducted. Two-tailed, unpaired Student’s *t*-tests were used to determine statistical significance when comparing two groups. A value of *p* < 0.05 was considered to be statistically significant.

## Results

### Lgr5+ HC Progenitors Generate More HCs Compared to the Lgr5- SCs *In Vitro*

**FIGURE 1:**
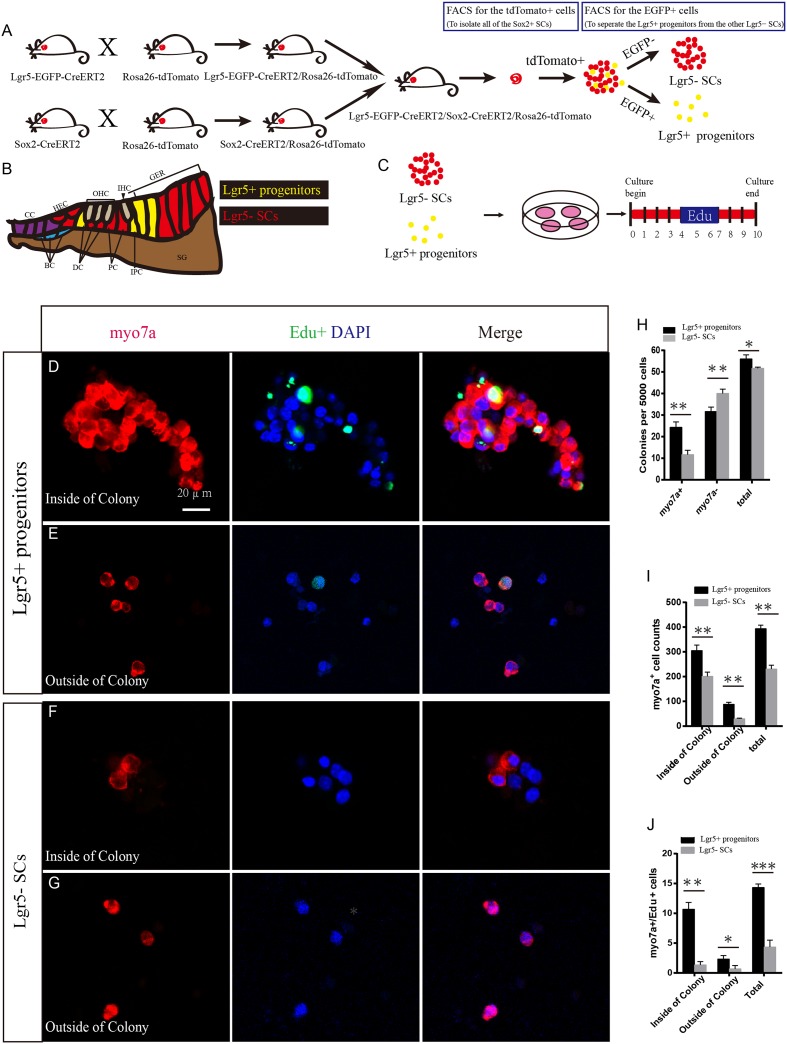
**Lgr5+ HC progenitors generate more HCs compared to Lgr5- SCs *in vitro.* (A)** We crossed the Lgr5-EGFP-CreERT2/Rosa26-tdTomato mice with Sox2-CreERT2/Rosa26-tdTomato mice to get the Lgr5-EGFP-CreERT2/Sox2-CreERT2/Rosa26-tdTomato triple-positive mice. We used the tdTomato channel to sort out all of the Sox2+ SCs, and then we used the FITC channel to separate the tdTomato/EGFP double-positive Lgr5+ progenitors from the other tdTomato+ but EGFP- SCs. Because Lgr5+ progenitors expressed both EGFP and tdTomato, the yellow dots represent the Lgr5+ progenitor cells. **(B)** Schematic depicting cell types in the P0–P3 cochlea. Lgr5+ progenitor cells were labeled in yellow and the Lgr5- SCs were labeled in red. DC, Deiters’ cells; PC, inner pillar cells; IPC, inner phalangeal cells; GER, the lateral greater epithelial ridge; BC, Boettcher cells; CC, Claudius cells; HEC, Hensen’s cells; SGN, spiral ganglion neuron. **(C)** We cultured the sorted EGFP+ cells at 50 cells/μl and added EdU from day 4 to 7. The total culture time was 10 days. **(D)** Lgr5+ progenitors generated a large number of Myo7a+ cells on the inside of the colony, and some of them were co-labeled with EdU. **(E)** Lgr5+ progenitors also generated some Myo7a+ cells outside of the colony. **(F,G)** Lgr5- SCs generated fewer Myo7a+ cells inside of the colony and outside of the colony, and few of them were co-labeled with EdU. **(H)** The number of colonies in each well per 5,000 cells. The Lgr5+ progenitors formed around 24 Myo7a+ colonies and 30 Myo7a- colonies, while the Lgr5- SCs formed around 11 Myo7a+ colonies and 40 Myo7a- colonies. **(I)** Both inside and outside of the colony, Lgr5+ progenitors formed more Myo7a+ cells compared with Lgr5- SCs. **(J)** In Lgr5+ progenitors, some of the Myo7a+ cells were co-labeled with EdU inside of the colony and outside of the colony (^∗^*p* < 0.05, ^∗∗^*p* < 0.01, ^∗∗∗^*p* < 0.001, *n* = 3. Scale bars are 20 μm in **D–G**).

Lgr5-EGFP-CreERT2 transgenic mice were used to report Lgr5 expression with EGFP (Hume et al., 2007). Lgr5-EGFP is expressed in a subset of SCs, including third-row Deiters’ cells, inner pillar cells, and inner phalangeal cells (Chai et al., 2011). To separate the Lgr5+ HC progenitors from the other Lgr5- SCs, we crossed Lgr5-EGFP-CreERT2/Rosa26-tdTomato mice with Sox2-CreERT2/Rosa26-tdTomato mice to generate Lgr5-EGFP-CreERT2/Sox2-CreERT2/Rosa26-tdTomato triple-positive mice among the offspring. Tamoxifen was injected at P2, and the cochleae were harvested at P4 (**Figure 1A**). Several previous studies have noted that the apical turn of the cochlea has greater HC regeneration ability than the basal turn (Chai et al., 2012; Cox et al., 2014), so to avoid the gene expression differences between the apical and basal turns (Waqas et al., 2016a) we used the middle turn of the cochlea for all of the experiments and analyses. For flow cytometry, we first used the tdTomato channel to sort out all the tdTomato+ cells (Red), which represent all of the Sox2+ SCs. From these tdTomato+ cells, we used the FITC channel to isolate the EGFP+ cells, which were the Lgr5+ progenitors, and the rest of the tdTomato+ but EGFP- cells were the Lgr5- SCs (**Figure 1A**). The Lgr5+ progenitors expressed both tdTomato and EGFP and thus were labeled in yellow in **Figure 1**. Using this strategy, we could separate the purified Lgr5+ progenitors from the other Lgr5- SCs (**Figure 1B**).

To determine the HC regeneration capability of Lgr5+ progenitors and Lgr5- SCs, we cultured 5,000 cells in laminin-coated 4-well dishes at a density of 50 cells/μl for 10 days in serum-free medium. We added 10 μM EdU to the culture medium from day 4 to 7 during culture to label the mitotically regenerated HCs (**Figure 1C**). After 10 days of culture, the cells were immunostained with the HC marker Myo7a. We found that the Lgr5+ progenitors generated significantly more Myo7a+ colonies and total colonies than the Lgr5- SCs (^∗∗^*p* < 0.01, ^∗∗∗^*p* < 0.001, *n* = 3) (**Figures 1D–H** and **Supplementary Figures S1A,B**), while the number of Myo7a- colonies was significantly greater for the Lgr5- SCs (^∗∗^*p* < 0.01, *n* = 3) (**Figure 1H**). Isolated Lgr5+ progenitor SCs generated HCs through both direct differentiation and mitotic regeneration. In the differentiation assay, the HCs inside of the colonies represent the mitotically regenerated HCs, and the HCs outside of the colonies represent the directly differentiated HCs. Next, we characterized and counted the Myo7a+ cells inside and outside of the epithelial colonies and found that Lgr5+ progenitors generated significantly more Myo7a+ HCs both inside and outside of the colony than the Lgr5- SCs (^∗^*p* < 0.05, ^∗∗^*p* < 0.01, *n* = 3) (**Figure 1I**). When we counted the Myo7a+/EdU+ cells, we found that the majority of the Myo7a+/EdU+ cells were inside of the colonies and only a few of the Myo7a+/EdU+ cells were outside of the colonies (**Figures 1D–G,J**) and that Lgr5+ progenitors generated significantly more Myo7a+/EdU+ HCs both inside and outside of the colonies than the Lgr5- SCs (^∗^*p* < 0.05, ^∗∗^*p* < 0.01, ^∗∗∗^*p* < 0.001, *n* = 3) (**Figure 1J**).

To further verify our findings, we used multiple HC markers, including Myo6 and parvalbumin (PV), to label the newly regenerated HCs. We found that all of the Myo7a+ cells were also Myo6+ and PV+ in both the population of HCs regenerated from Lgr5+ progenitors and the population generated from Lgr5- SCs (**Supplementary Figures S1C–F**). To further investigate the bundle morphology of newly regenerated HCs, we used the common hair bundle markers phalloidin and espin1. We found that 68.7 and 66.3% of newly regenerated HCs had hair bundles from Lgr5+ progenitors and Lgr5- SCs, respectively (**Supplementary Figures S1E,F’,G,H**). To test whether the newly regenerated HCs have mechanosensory transduction function, we performed additional FM1-43 dye experiments. Almost all of the newly regenerated HCs from both Lgr5+ progenitors and Lgr5- SCs could take up FM1-43 dye, suggesting that the majority of the newly regenerated HCs had the mechanosensory transduction function (**Supplementary Figures S1I,J**). Taken together, these results suggest that Lgr5+ progenitors generate significantly more HCs than the Lgr5- SCs *in vitro*.

### Lgr5+ Progenitors have Higher Sphere-Forming Ability than Lgr5- SCs

**FIGURE 2:**
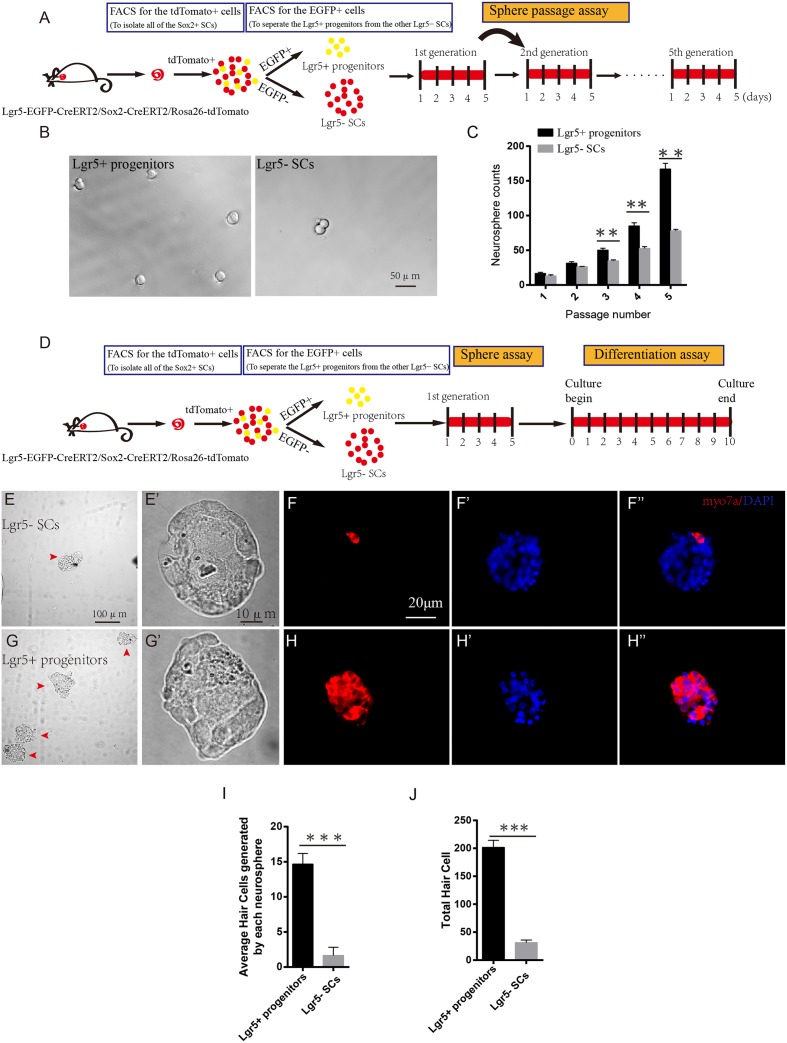
**Lgr5+ progenitors have greater sphere-forming ability than Lgr5- SCs. (A)** Tamoxifen was injected at P3, and the mice were harvested at P5. FACS was used to isolate the Lgr5+ progenitors and Lgr5- SCs, and these cells were cultured for 5 days and passaged to the next generation. **(B)** Lgr5+ progenitors generated significantly more spheres than Lgr5- SCs. **(C)** Lgr5+ progenitors had a significantly higher rate of expansion than Lgr5- SCs. **(D)** The cultured cells in the first generation were used for the differentiation assay. **(E,E’)** The Differential Interference Contrast microscope configuration (DIC) pictures show the low magnification **(E)** and high magnification **(E’)** images of the spheres formed by Lgr5- SCs. **(G,G’)** The DIC pictures show the low magnification **(G)** and high magnification **(G’)** images of the spheres formed by Lgr5+ progenitors. **(F,F”)** An Lgr5- sphere stained with the HC marker Myo7a. **(F’)** Represents the sphere stained with DAPI, **(F”)** represents merged image. **(H,H”)** An Lgr5+ sphere stained with the HC marker Myo7a. **(H’)** Represents the sphere stained with DAPI and **(H”)** shows the merged image. **(I)** The average number of HCs generated by each sphere. **(J)** The total number of hair cells generated by 200 Lgr5+ progenitors or Lgr5- SCs. ^∗^*p* < 0.05, ^∗∗^*p* < 0.01, ^∗∗∗^*p* < 0.001, *n* = 3. Scale bars are 20 μm in **(E,F)**.

Sphere-forming assays have been used to evaluate cell proliferation in many studies (Li et al., 2003; Sinkkonen et al., 2011; Chai et al., 2012; Shi et al., 2012; Jan et al., 2013; Lu et al., 2016). To determine the sphere-forming ability of Lgr5+ progenitors and Lgr5- SCs, we purified the two cell populations by flow cytometry, cultured 200 sorted cells at a density of 2 cells/μl in a Costar ultra-low attachment plate for 5 days, and counted the number of spheres before passage to the next generation (**Figure 2A**). We found that Lgr5+ progenitors formed significantly more spheres than the Lgr5- SCs (*p* < 0.05, *n* = 3), but there was no difference in the size of the spheres (**Figures 2B,C**). To further evaluate the HC regeneration ability of these spheres, we isolated the spheres derived from Lgr5+ progenitors and the Lgr5- SCs from the first generation and differentiated the spheres for 10 days. EdU was added from day 4 to 7 during the culture, and the cells were stained for Myo7a after culture (**Figure 2D**). We counted the Myo7a+ HCs in each differentiated sphere as well as the total number of Myo7a+ HCs. We found that each sphere that originated from Lgr5+ progenitors generated almost 8 times as many Myo7a+ HCs than spheres from the Lgr5- SCs (**Figures 2E–H**), and the total spheres originating from Lgr5+ progenitors gave rise to significantly more total Myo7a+ HCs than those originating from the Lgr5- SCs (**Figures 2I,J**). In a similar manner, we used Myo6 and PV to label the newly regenerated HCs, and we found that all of the Myo7a+ cells were also Myo6+ and PV+ in both groups (**Supplementary Figures S2A–D**). We also used phalloidin and espin1 to show the bundle morphology of newly regenerated HCs, and we found that 61.4 and 58.3% of newly regenerated HCs had hair bundles from Lgr5+ progenitors and Lgr5- SCs, respectively (**Supplementary Figures S2A’,B’,G,H,G’,H’**). We also performed the FM1-43 dye experiments to test whether the newly regenerated HCs had the mechanosensory transduction function. We found that almost all of the newly regenerated HCs from both groups could take up FM1-43 dye, suggesting that the majority of the newly regenerated HCs had the mechanosensory transduction function (**Supplementary Figures S2E,F**). These results showed that Lgr5+ progenitors had a greater ability to proliferate and to generate HCs than the other Lgr5- SCs.

### Analysis of RNA-Seq Results

**FIGURE 3:**
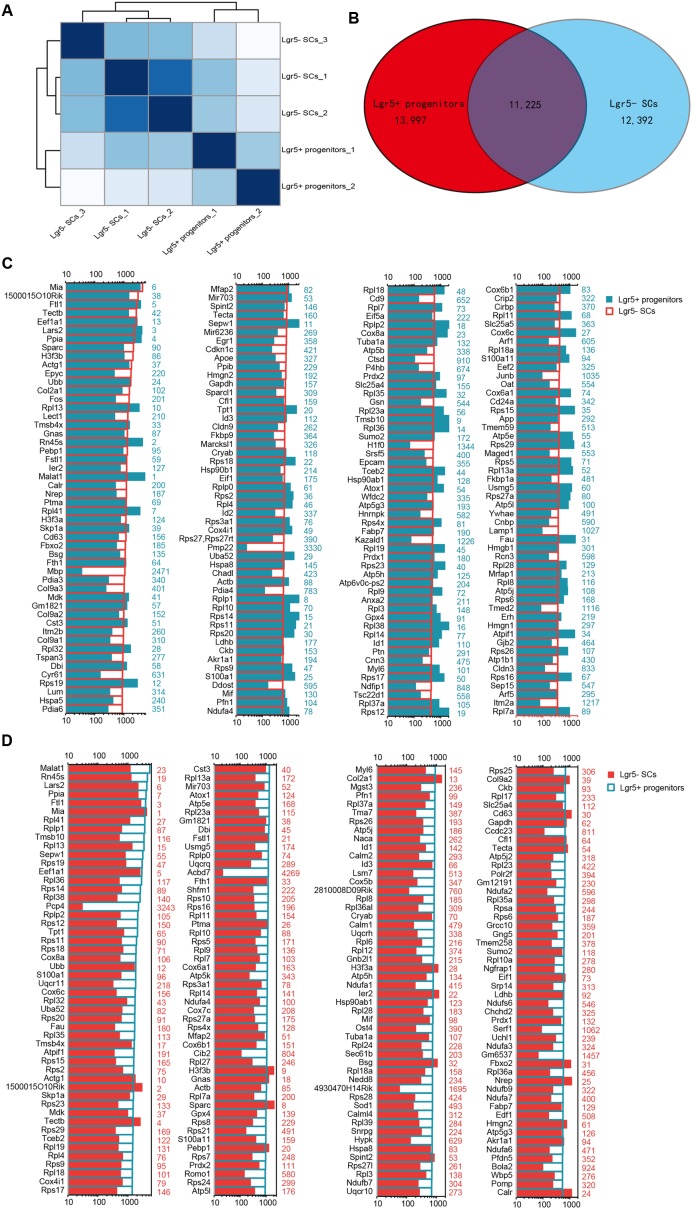
**Top 200 genes highly expressed in Lgr5+ progenitors and Lgr5- SCs. (A)** Sample clustering analysis for all replicates of Lgr5+ progenitors and Lgr5- SCs. **(B)** Venn diagram showing genes expressed in Lgr5+ progenitors and Lgr5- SCs. **(C)** The top 200 genes that are highly expressed in Lgr5- SCs ranked in descending order. The number in blue on the right side of each panel represents the same gene ranking in Lgr5+ progenitors. **(D)** The top 200 genes highly expressed in Lgr5+ progenitors ranked in descending order. The number in red on the right side of each panel represents the same gene ranking in Lgr5- SCs.

We performed RNA-Seq analysis to identify differences in gene expression between Lgr5+ progenitors and Lgr5- SCs. Between 30.8 and 47.7 million paired-end reads were obtained for each sample, with 58.1–75.5% of the read pairs mapping correctly to the reference genome (mouse mm9). The expression of every gene was measured by FPKM (Fragments Per Kilobase of transcript per Million fragments mapped), and we filtered out genes with low expression (FPKM < 1). Three replicates of each population showed high reproducibility (Pearson’s *r* = 0.923–0.957 for the Lgr5- SC populations and 0.941 for the Lgr5+ progenitor populations) (**Figure 3A**). After excluding genes with FPKM below the baseline, 13,997 and 12,392 genes were expressed in the Lgr5+ progenitors and the Lgr5- SCs, respectively, and 11,225 of these genes were expressed in both cell populations (**Figure 3B**).

### Genes Enriched in Lgr5+ Progenitors or Lgr5- SCs

In order to characterize the gene-expression profiles in Lgr5+ progenitors and Lgr5- SCs, we explored the most abundantly expressed genes in both populations. **Figure 3C** shows the expression levels for the top 200 most abundant genes in Lgr5- SCs. For comparison, expression levels for the same transcripts in the Lgr5+ progenitors and abundance rankings for these transcripts are also illustrated. **Figure 3D** similarly shows the 200 most abundant transcripts in Lgr5+ progenitors compared to the same transcripts in Lgr5- SCs. As shown in both figures, the majority of the transcripts that are richly expressed in one population are also abundantly expressed in the other. However, among the most abundantly expressed genes, *Mbp*, *Pmp22*, and *H1f0* were significantly highly expressed in Lgr5- SCs, and *Pcp4*, *Acbd7*, *4930170H14Rik*, and *Gm6537* were significantly highly expressed in Lgr5+ progenitors. None of these genes have been previously reported to be expressed in the inner ear.

### Differentially Expressed Genes in Lgr5+ Progenitors and Lgr5- SCs

**FIGURE 4:**
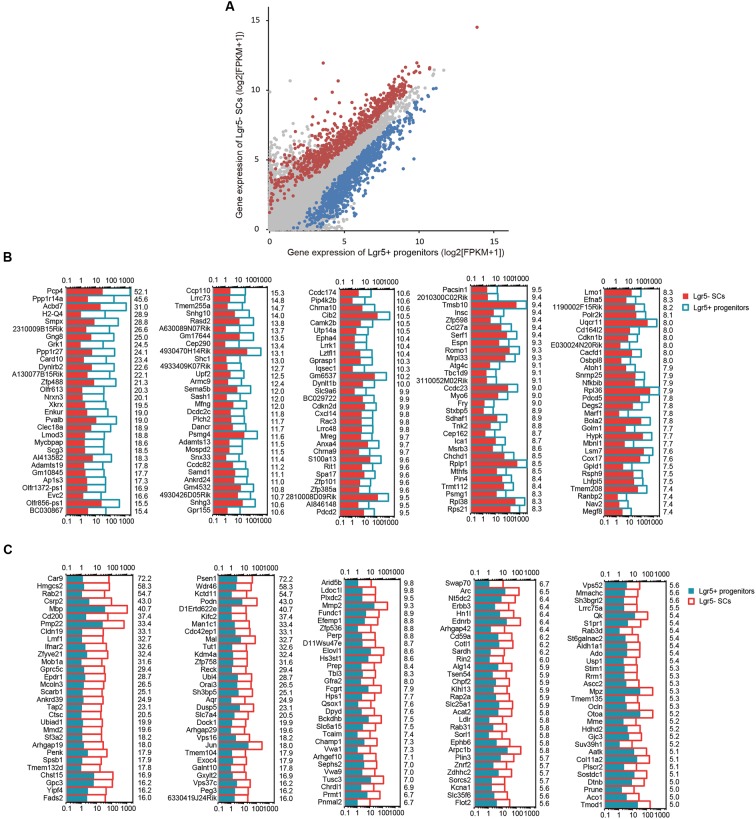
**The differentially expressed genes in Lgr5+ progenitors and Lgr5- SCs. (A)** All expressed transcripts in Lgr5- SCs and Lgr5+ progenitors. The blue dots represent the highly differentially expressed genes in Lgr5+ progenitors, the red dots represent the highly differentially expressed genes in Lgr5- SCs, and the gray dots represent the genes that are highly expressed in both Lgr5- SCs and Lgr5+ progenitors. **(B)** The top 150 highly differentially expressed genes in Lgr5+ progenitors ranked in descending order. The number on the right of each panel represents the fold difference in expression for Lgr5+ progenitors versus Lgr5- SCs. **(C)** The top 150 highly differentially expressed genes in Lgr5- SCs ranked in descending order. The number on the right of each panel represents the fold difference in expression for Lgr5- SCs versus Lgr5+ progenitors.

To determine which genes are differentially expressed in Lgr5+ progenitors and Lgr5- SCs, we compared the expression levels of all of the transcripts in Lgr5+ progenitors with those of Lgr5- SCs and selected the top differentially expressed genes (**Figure 4A**). Differentially expressed genes were categorized as those whose expression levels were above background and at least 2-fold different between the Lgr5+ progenitors and Lgr5- SCs (*p* < 0.05). We found 1,826 genes that were differentially highly expressed in Lgr5- SCs and 986 genes that were differentially highly expressed in Lgr5+ progenitors. **Figures 4B,C** show the top 150 differentially expressed genes in Lgr5+ progenitors and Lgr5- SCs. The functions of some of the differentially expressed genes have been reported previously. Some of the genes that are highly expressed in Lgr5+ progenitors have been reported to play roles in inner ear HC development, ear morphogenesis, and neuron projection, including *Cib2* (Ahmed et al., 2013), *Epha4* (Defourny et al., 2013), *Espn* (Sekerkova et al., 2006), *Lhfpl5* (Zhao et al., 2014), *Smpx* (Huebner et al., 2011; Schraders et al., 2011), and *Lmo1* (Deng et al., 2006), and this supports our notion that Lgr5+ progenitors have a much greater potential to generate more sensory HCs in the neonatal cochlea. However, a significant number of the differentially expressed genes have not been characterized before and need to be further studied in the future.

### Cell Cycle Analysis

**FIGURE 5:**
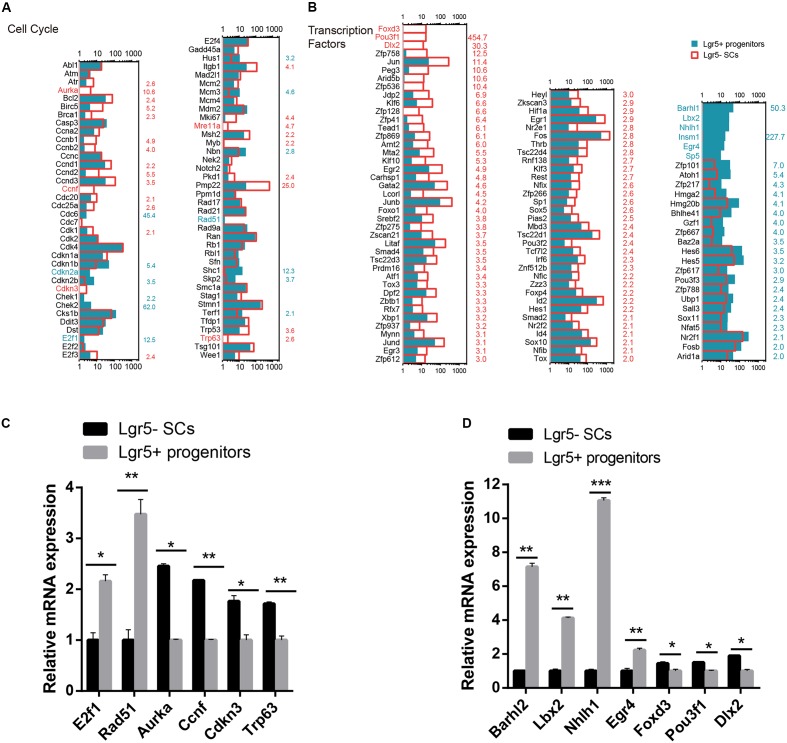
**Cell cycle genes and transcription factors in Lgr5- SCs and Lgr5+ progenitors. (A)** The expression of 80 genes involved in the cell cycle in Lgr5- SCs and Lgr5+ progenitors. **(B)** The expression of 83 transcription factor genes in Lgr5- SCs and Lgr5+ progenitors. In **(A,B)**, the gene names in red on the left of each panel represent the genes that are uniquely detected in Lgr5- SCs, and the blue names represent the genes that are uniquely detected in Lgr5+ progenitors. The number in red on the right of each panel represents the fold difference in expression for Lgr5- SCs versus Lgr5+ progenitors, and the blue number on the right of each panel represents the fold difference in expression for Lgr5+ progenitors versus Lgr5- SCs. **(C)** q-PCR analysis of the cell cycle genes. **(D)** q-PCR analysis of the transcription factors. ^∗^*p* < 0.05, ^∗∗^*p* < 0.01, ^∗∗∗^*p* < 0.001, *n* = 3.

Lgr5+ progenitors had significantly greater proliferation and mitotic HC regeneration ability than the other Lgr5- SCs; however, the detailed mechanism behind this difference remains unknown. To identify the possible genes regulating the cell cycling of Lgr5+ progenitors, we used RNA-Seq analysis to compare the expression of genes regulating the cell cycle and cell proliferation in Lgr5+ progenitors and Lgr5- SCs. It has been reported that over 1,000 cell cycle genes might exist in the mammalian cell (Forrest et al., 2003), and we examined the expression of 80 genes known to be involved in the cell cycle and that are commonly assayed in cell cycle PCR arrays. We found that *Cdkn1b*, *Cdkn2a*, *E2f1*, *Rad51*, and *Shc1* were significantly highly expressed in Lgr5+ progenitors and that *Aurka*, *Bcl2*, *Ccnd1*, *Ccnd*2, *Ccnd3*, *Ccnf*, *Cdkn3*, *Itgb1*, *Mki67*, *Mre11a*, *Msh2*, *Pmp22*, and *Trp63* were significantly highly expressed in Lgr5- SCs (**Figure 5A**). However, most of the differentially expressed cell cycle-regulating genes that we identified in Lgr5+ progenitors and Lgr5- SCs have not been characterized before in the inner ear and need to be further studied. We performed q-PCR to confirm the RNA-Seq data, and the results were consistent with the microarray analysis data (**Figure 5C**). We did not detect the expression of *Aurka* or *Mre11a*, possibly because of their low gene expression.

### Transcription Factors Analysis

Transcription factors (TFs) are proteins that bind to either enhancer or promoter regions of genes thereby controlling the expression level of these target genes. TFs are involved in various processes, including inner ear development and HC regeneration. To determine which TFs might be involved in regulating HC regeneration, we examined the expression of 1,324 TFs in the mouse genome in Lgr5+ progenitors and Lgr5- SCs. **Figure 5B** shows the 83 significantly differentially expressed TFs in Lgr5+ progenitors and Lgr5- SCs (*p* < 0.05, fold change > 2). We found that the genes for six TFs (*Barhl1, Lbx2, Nhlh1, Insm1, Egr4, and Sp5*) were richly expressed in Lgr5+ progenitors but not detected in the Lgr5- SCs at all. Some of the TF genes that were highly expressed in Lgr5+ progenitors have been reported to play roles in promoting HC fate and patterning regulation during inner ear development, including *Atoh1*, *Barhl1*, *Hmga2*, *Pou3f3*, and *Sox11* (Mutai et al., 2009; Chonko et al., 2013; Smeti et al., 2014; Gnedevaa and Hudspeth, 2015), and some of the TF genes that are highly expressed in Lgr5- SCs have been reported to play critical roles in regulating cell survival and apoptosis in the inner ear, including *Gata2* (Haugas et al., 2010), *Hif1a* (Chung et al., 2011), *Thrb* (Ng et al., 2001), *Jun* (Sanz et al., 1999), *Smad4* (Yang et al., 2009), and *Hes1* (Kelley, 2006). We performed q-PCR to confirm the RNA-Seq data, and the results were consistent with the microarray analysis data (**Figure 5D**). We did not detect the expression of *Sp5*, possibly as a result of the low gene expression. We have identified many TFs that have not been characterized before, and their involvement in regulating the HC regeneration capacity of Lgr5+ progenitors and Lgr5- SCs should be investigated in the future.

### Signaling Pathway Analysis

**FIGURE 6:**
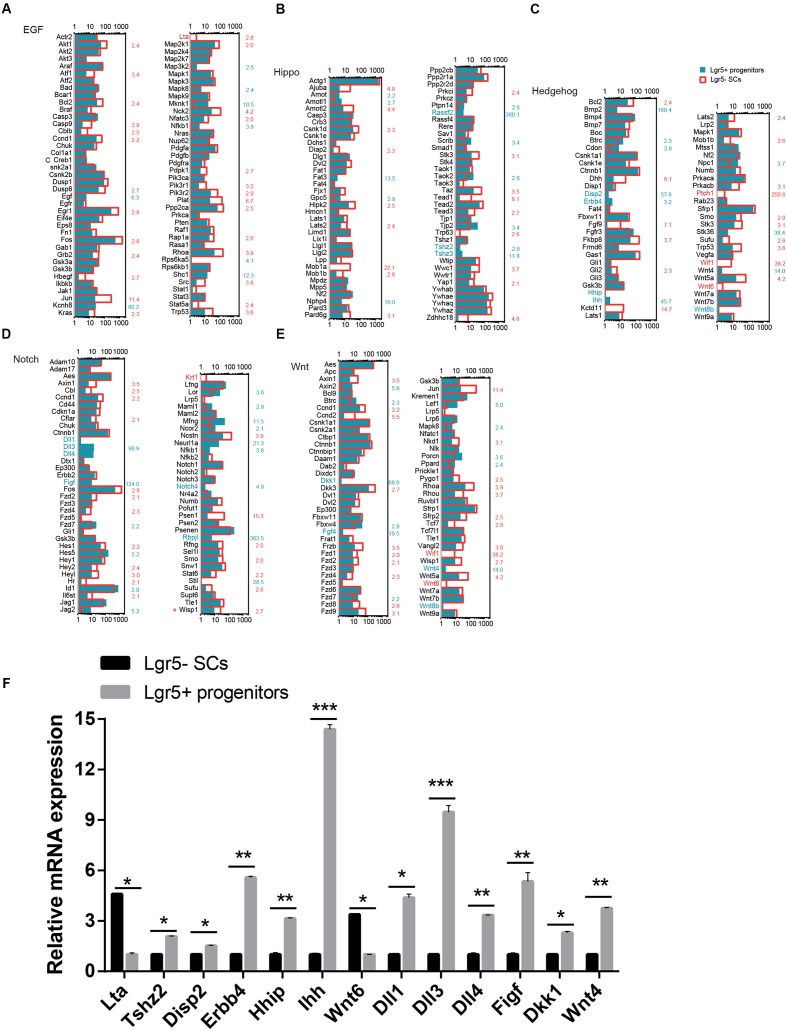
**Signaling pathway genes in Lgr5- SCs and Lgr5+ progenitors**. The differentially expressed genes in Lgr5- SCs and Lgr5+ progenitors that are involved in **(A)** EGF, **(B)** Hippo, **(C)** Hedgehog, **(D)** Notch, and **(E)** Wnt signaling pathways. The gene names in red on the left of each panel represent the genes uniquely detected in Lgr5- SCs, and the names in blue represent the genes uniquely detected in Lgr5+ progenitors. The number in red on the right of each panel represents the fold difference in expression for Lgr5- SCs versus Lgr5+ progenitors, and the number in blue on the right of each panel represents the fold difference in expression for Lgr5+ progenitors versus Lgr5- SCs. **(F)** q-PCR analysis of the EGF, Hippo, Hedgehog, Notch, and Wnt signaling pathway genes. ^∗^*p* < 0.05, ^∗∗^*p* < 0.01, ^∗∗∗^*p* < 0.001, *n* = 3.

Several major signaling pathways play important roles in regulating cell proliferation and HC regeneration, including the EGF, Hedgehog, Wnt, Notch, and Hippo pathways. To determine which signaling pathway factors are involved in regulating the proliferation and HC regeneration ability of Lgr5+ progenitors and Lgr5- SCs, we measured over 1000 genes, many of which had significant differences in expression (**Figure 6**). The pathway genes that are highly expressed in Lgr5+ progenitors include *Kcnh8*, *Mknk1*, *Shc1*, *Cdon*, *Disp2*, *Erbb4*, *Hhip*, *Ihh*, *Npc1*, *Dll1*, *Dll3*, *Dll4*, *Figf*, *Hes5*, *Mfng*, *Neurl1a*, *Notch4*, *Rbpjl*, *Dkk1*, *Fgf4*, *Wnt4*, *Wnt8b*, *Fat3*, *Rassf2*, *Tjp2*, *Tshz2*, and *Tshz3*. Among them, *Axin2* (Jan et al., 2013; Jansson et al., 2015), *Egf* (Lou et al., 2015), *Wnt4* (Alvarado et al., 2011), *Tjp2* (Kim et al., 2014), *Dll1* (Kiernan et al., 2005; Chrysostomou et al., 2012), and *Dll3* (Hartman et al., 2007) have already been reported in the inner ear. Some of the pathway genes that are highly expressed in Lgr5- SCs include *Akt1*, *Atf1*, *Bcl2*, *Ccnd1*, *Dusp6*, *Egr1*, *Fos*, *Grb2*, *Jun*, *Kras*, *Lta*, *Pik3r1*, *Pik3r2*, *Plat*, *Ppp2ca*, *Rhoa*, *Dhh*, *Fgf9*, *Fkbp8*, *Kctd11*, *Prkacb*, *Ptch1*, *Stk3*, *Fos*, *Hesl*, *Heyl*, *Krt1*, *Ncstn*, *Psen1*, *Ccnd2, Dkk3, Fzd8, Fzd9, Fkbp8, Jun, Nkd1, Rhou*, *Wif1*, *Frzb*, *Wnt5a, Wnt6*, *Ajuba*, *Amotl2*, *Csnk1d*, *Hipk2*, *Mob1a*, *Pard6g*, *Stk3*, *Wisp1*, *Taz*, *Tead1*, and *Wwc1*. Among these, *Dusp6* (Urness et al., 2008), *Rhoa* (Sai et al., 2014), *Fgf9* (Huh et al., 2015), *Frzb* (Qian et al., 2007), *Fzd1*, *Fzd4*, *Fzd9* (Shah et al., 2009), *Fkbp8* (Zak et al., 2011), *Hey2* (Benito-Gonzalez and Doetzlhofer, 2014), *Src* (Andreeva et al., 2014), *Smo* (Tateya et al., 2013), *Vangl2* (Copley et al., 2013), *Wnt5a* (Qian et al., 2007), *Wnt6* (Lillevali et al., 2006), and *Wif1* (Dabdoub et al., 2003) have already been reported in the inner ear. Most of the cell-signaling pathway genes have not been characterized before in the inner ear.

We performed q-PCR to confirm the RNA-Seq data, and the result was consistent with the microarray analysis data (**Figure 6F**). We did not detect the expression of *Wnt8*, *Ptch1*, *Wif1*, *Krt1*, or *Fgf4*, possibly as a result of their low gene expression. The different expression of these genes might be involved in regulating the different proliferation and regeneration ability of Lgr5+ progenitors compared to the Lgr5- SCs.

### Gene Ontology Analysis of the Differentially Expressed Genes in Lgr5+ Progenitors and Lgr5- SCs

**FIGURE 7:**
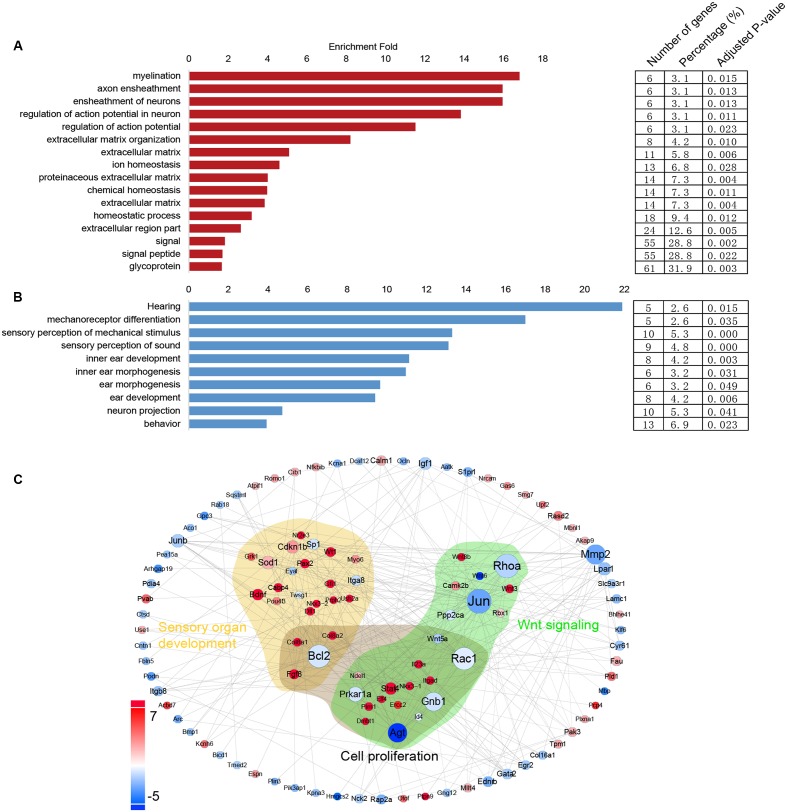
**Gene ontology and network analysis of the genes differentially expressed in Lgr5+ progenitors and Lgr5- SCs and the PCA analysis. (A)** The functions of genes upregulated in Lgr5- SCs. **(B)** The function of genes upregulated in Lgr5+ progenitors. **(C)** The STRING protein–protein interaction analysis of genes that are differentially expressed in Lgr5+ progenitors (blue) and Lgr5- SCs (red).

In order to obtain a comprehensive view of the gene network involved in inner ear HC regeneration, we performed a STRING protein–protein interaction analysis (Franceschini et al., 2013), which assembles the predicted networks of the differentially expressed genes (fold change > 2.0, *p* < 0.01) with the functional categories highlighted by GO analysis (DAVID) (**Figure 7C**). This integrated GO analysis suggests a complex network of genes that are involved in inner ear HC development and are predicted to participate in regulating inner ear development, cell proliferation, and Wnt signaling. GO analysis was also applied to the genes that are upregulated in Lgr5+ progenitors and Lgr5- SCs (fold change > 2.0, *p* < 0.01) (**Figures 7A,B**). As shown in **Figure 7A**, genes upregulated in Lgr5+ progenitors were highly enriched in functional categories such as hearing, mechanoreceptor differentiation, and inner ear development, while the genes upregulated in Lgr5- SCs were slightly enriched in functional categories such as signaling and the extracellular matrix.

## Discussion

In the mouse inner ear, SCs can divide and transdifferentiate into HCs (White et al., 2006; Cox et al., 2014; Lu et al., 2016). Lgr5 is only expressed in a subset of SCs, and it is enriched in the population of HC progenitors (Chai et al., 2011; Shi et al., 2012). The Lgr5+ progenitors have a greater capacity to regenerate HCs both *in vitro* and *in vivo* than Lgr5- SCs, and Lgr5+ progenitors can be regulated by Wnt and Notch signaling to regenerate HCs via both direct differentiation and mitotic regeneration (Wang et al., 2015; Li et al., 2016; Lu et al., 2016; Ni et al., 2016; Waqas et al., 2016b). When isolated by FACS, the Lgr5+ progenitors can be passaged for at least five generations (Chai et al., 2012; Shi et al., 2012, 2013; Bramhall et al., 2014; Li et al., 2016; Waqas et al., 2016a; Wu et al., 2016). In this study, we isolated the Lgr5+ progenitors and the other Lgr5- SCs from transgenic mice by flow cytometry. The Lgr5+ progenitors differentiated to form more Myo7a+ HCs, and they formed more spheres than Lgr5- SCs. To understand the mechanism behind the different proliferation and HC regeneration ability of Lgr5+ progenitors compared to the other Lgr5- SCs, we determined the genome-wide transcriptional profiles of these two cell populations via RNA-Seq profiling.

### Differentially Expressed Genes in Lgr5+ Progenitors and Lgr5- SCs

Among the top 150 differentially expressed genes, most of them have not been reported in the inner ear, and only a few of them have been described before. The genes that are highly expressed in Lgr5+ progenitors include *Cib2*, *Epha4*, *Espn*, *Atoh1*, *Lhfpl5*, *Smpx*, and *Lmo1*. *Epha4* is expressed in outer HCs and spiral ganglion neurons (SGNs), and it mediates afferent signaling to HCs (Defourny et al., 2013). *Lhfpl5* mutation affects tip-link assembly (Zhao et al., 2014), and *Smpx* is strongly expressed in the sensory epithelium and plays a role in HC formation (Huebner et al., 2011; Schraders et al., 2011). *Lmo1* is suggested to play an important role in HC differentiation and is specifically expressed in cochlear HCs and vestibular HCs during the development of the inner ear (Deng et al., 2006). The genes *Cib2* and *Espn* are involved in the formation of stereocilia in the inner ear, and their disruption can lead to hearing impairment (Sekerkova et al., 2006; Ahmed et al., 2013). Our analysis showed that some of the genes that are highly expressed in Lgr5+ progenitors are crucial for HC formation during inner ear development, and expression of these genes might be the source of the high HC regeneration capabilities of Lgr5+ progenitors.

The set of reported genes that are highly expressed in Lgr5- SCs includes *Ednrb*, *S1pr1*, and *Tekt2*. *Ednrb* mutation causes syndromic hearing loss due to congenital defects in the melanocytes in the stria vascularis of the inner ear (Matsushima et al., 2014). *S1pr1* is expressed in both the organ of Corti and the SGN, and it plays a role in maintaining the function of vestibular and cochlear HCs (Nakayama et al., 2014). *Tekt2* is also expressed in HCs and participates in the transient appearance of the microtubule-based kinocilium in the cochlear HCs (Yoon et al., 2011). The genes that are highly expressed in Lgr5- SCs are mainly involved in the function of the cochlea.

Furthermore, we analyzed the cell cycle genes, TF genes, and signaling pathway factor genes that might regulate proliferation and HC regeneration ability, and we found 8 cell cycle genes, 9 TF genes, and 24 signaling pathway factor genes that are uniquely expressed in either Lgr5+ progenitors or Lgr5- SCs.

### Cell Cycle Analysis

The highly expressed genes in Lgr5+ progenitors include *E2f1*, *Cdkn1b*, and *Rad51*. *E2f1* is expressed in the SGN, and mitochondrial reactive oxygen species-mediated *E2f1* activation induces apoptosis in the SGN (Raimundo et al., 2012). The *Cdkn1b* and *Rad51* genes in the auditory sensory epithelium promote the proliferation and formation of supernumerary HCs in the post-natal and adult cochlea (Chen and Segil, 1999; Lowenheim et al., 1999; Oesterle et al., 2014; Walters et al., 2014).

The genes that are highly expressed in Lgr5- SCs include *Bcl2*, *Birc5*, *Pkd1*, *Trp63*, and *Itgb1*. *Bcl2* knockout mice have high-frequency hearing loss due to a developmental defect in the stapes (Carpinelli et al., 2012; Liu et al., 2014). *Birc5* is broadly expressed in the cochlea, and it can protect HCs against damage (Habtemichael et al., 2010). *Pkd1* is localized to the HC stereocilia, and it plays an essential role in stereocilia structure and maintenance (Steigelman et al., 2011). *Trp63* is important for normal development of the cochlea by activating the Notch signaling pathway (Terrinoni et al., 2013). *Itgb1* is expressed throughout the otic area – including the sensory epithelium and the periodic mesenchyme – during inner ear development (Matilainen et al., 2007). We also found other cell cycle-promoting genes (including *Ran*, *Stmn1*, and *Smc1a*) and cell cycle-inhibiting genes (including *E2f4*, *Rbl1*, and *Mdm2*) that are abundantly expressed in both cell populations. In addition to these, the other newly identified cell cycle regulatory genes in Lgr5+ progenitors and Lgr5- SCs need to be further characterized.

### Transcription Factor Analysis

The highly expressed TF genes in Lgr5+ progenitors include *Barhl1*, *Atoh1*, *Hmga2*, *Pou3f3*, and *Sox11*. Atoh1 promotes cochlear HC survival and differentiation, and *Barhl1* is a downstream gene of *Atoh1* that is essential for HC maintenance (Chonko et al., 2013). *Hmga2* is broadly expressed during inner ear development, which suggests its potential dual role in early differentiation and in the maintenance of both HC and SC phenotypes (Smeti et al., 2014). *Pou3f3* is specifically expressed in SCs and mesenchymal cells, and it is important for the maintenance and functional development of the post-natal cochlea (Mutai et al., 2009). *Sox11* promotes the differentiation of SCs into HCs (Gnedevaa and Hudspeth, 2015).

The TF genes that were highly expressed in Lgr5- SCs include *Gata2*, *Hif1a*, *Thrb*, *Jun*, *Smad4*, and *Hes1*. *Gata2* is required for vestibular morphogenesis (Haugas et al., 2010). *Hif1a* and *Thrb* are expressed in HCs, and high expression of *Hif1a* prevents noise-induced hearing loss (Chung et al., 2011), while a lack of *Thrb* leads to the developmentally delayed establishment of potassium currents (Ng et al., 2001). The *Jun* gene plays a critical role during inner ear development by mediating apoptosis through the JNK pathway (Sanz et al., 1999). *Smad4* is required for inner ear development (Yang et al., 2009), and *Hes1* inhibits SC differentiation by decreasing the expression of Atoh1 (Kelley, 2006). Our results suggest that the higher expression of these negative transcriptional regulators might be involved in the reduced proliferation capacity of Lgr5- SCs compared to Lgr5+ progenitors

### Signaling Pathway Analysis

The signaling factor genes that are highly expressed in Lgr5+ progenitors include *Axin2, Wnt4, Tjp2, Dll1*, and *Dll3. Axin2* acts as a Wnt target gene, and its expression in tympanic border cells allows them to behave as HC progenitors (Jan et al., 2013; Jansson et al., 2015). Combined with other growth factors, *Egf* can protect HCs from ototoxic damage (Lou et al., 2015). *Wnt4* is detected in the inner and outer spiral sulcus cells, as well as in the Claudius and Hensen’s cells, and downregulation of *Wnt4* expression significantly reduces the proliferation of SCs (Alvarado et al., 2011). The *Tjp2* gene is mainly expressed in the membrane between the HCs and SCs, and mutation of the *Tjp2* gene causes hearing loss (Op de Beeck et al., 2011; Kim et al., 2014). Both *Dll1* and *Dll3* can repress HC formation and can promote HC differentiation (Kiernan et al., 2005; Hartman et al., 2007; Chrysostomou et al., 2012; Petrovic et al., 2014).

The signaling factor genes that are highly expressed in Lgr5- SCs include *Dusp6*, *Egf*, *Rhoa*, *Fgf9*, *Frzb*, *Fzd1*, *Fzd4*, *Fzd9*, *Fkbp8*, *Hey2*, *Src*, *Smo*, *Vangl2*, *Wnt5a*, *Wnt6*, and *Wif1*. *Dusp6* is expressed in the otic region during embryonic development and acts as a negative feedback regulator of FGF signaling (Urness et al., 2008). *Rhoa*, *Wnt5a*, *Src*, *Vangl2*, and *Wif1* mediate planar cell polarity in the inner ear (Dabdoub et al., 2003; Qian et al., 2007; Copley et al., 2013; Andreeva et al., 2014; Sai et al., 2014), and *Src* inhibitors protect HCs from noise-induced damage (Bielefeld, 2015). *Fgf9* participates in regulating the number of cochlear progenitors and the length of the cochlea through mesenchymal FGFR signaling (Pirvola et al., 2004; Huh et al., 2015). *Smo* is an effector of Hedgehog signaling that inhibits prosensory cell differentiation into HCs or SCs and delays differentiation in the apical region (Tateya et al., 2013). *Fzd1*, *Fzd4*, and *Fzd9* are expressed in adult mouse SGNs, and *Fzd9* guides neurite regeneration in SGNs (Shah et al., 2009). *Frzb* is expressed in the lateral region of the developing organ of Corti and regulates stereociliary orientation and cochlear extension (Qian et al., 2007). *Fkbp8* is localized in the SGN and is important for the onset of hearing processes in rodents (Zak et al., 2011). *Hey2* is highly expressed in HCs and SCs, and it is critical for maintaining prosensory cells in an undifferentiated state (Benito-Gonzalez and Doetzlhofer, 2014). *Wnt6* is the first Wnt gene expressed in the otic epithelium at embryonic day 8.5, and its expression is confined to the dorsal portion of the otic placode (Lillevali et al., 2006). Our analysis shows that the genes that are highly expressed in Lgr5- SCs are mainly involved in the function of the cochlea but not in cell proliferation or HC regeneration.

### STRING Prediction of Inner Ear HC Development

In this protein–protein interaction network, most of the genes in the GO categories of sensory organ development were highly expressed in Lgr5+ progenitors, such as *Gfi1*, *Pou4f3*, and *Pax2*, although several genes such as *Eya1* and *Bcl2* were richly expressed in Lgr5- SCs. It would be interesting to further investigate the involvement of these genes in regulating the progenitor cells.

All of the data we provided in this paper were from the neonatal mouse cochlea. The situation might be completely different in the adult mouse cochlea or in the damaged mouse cochlea, and the approaches to promote HC regeneration in the neonatal cochlea might not have the same effects in the adult cochlea. Thus, further investigations in the adult cochlea or damaged cochlea need to be performed in the future.

## Conclusion

We found that Lgr5+ progenitors have significantly greater proliferation and HC regeneration ability than Lgr5- SCs. We investigated the transcriptome differences between Lgr5+ progenitors and Lgr5- SCs and found significantly differentially expressed genes that might regulate the Lgr5+ progenitors’ proliferation and HC regeneration capacity. The most interesting of these are the genes that are uniquely expressed in Lgr5+ progenitors but not in Lgr5- SCs. To further analyze the role of differentially expressed genes in HC regeneration and proliferation, we constructed a STRING prediction map. The transcriptomes of Lgr5+ progenitors and Lgr5- SCs reported here establish a framework for future characterization of the genes that regulate the proliferation and HC regeneration ability of Lgr5+ progenitors, and these genes might represent new therapeutic targets for HC regeneration.

## Author Contributions

CC, LG, LL, HS, HL, and RC designed the study. CC, LG, HS, XX, and SZ performed the laboratory experiments. RC, CC, LG, HS, CX, HL, MW, YC, FC, XZ, XG, and MT contributed to critical discussion and data analysis. CC, MW, HS, RC, HL, JG, and LG wrote the paper. All authors read and approved the final manuscript.

## Conflict of Interest Statement

The authors declare that the research was conducted in the absence of any commercial or financial relationships that could be construed as a potential conflict of interest.

## Supplementary Material

FIGURE S1 **Lgr5+ HC progenitors generate more HCs compared to Lgr5- SCs *in vitro*. (A,B)** After 10 days of culture, the low magnification images of the inside colonies and outside colonies showed that Lgr5+ progenitors generated more Myo7a+ colonies than Lgr5- SCs. **(C,D)** The HC marker Myo6 labeled the newly regenerated HCs in Lgr5- SCs and Lgr5+ progenitors. All the Myo7a+ cells are also Myo6+ in both the population of HCs regenerated from Lgr5+ cells and the population regenerated from Lgr5- SCs. **(E,F)** The HC marker PV also labeled the newly regenerated HCs in Lgr5- SCs and Lgr5+ progenitors. All of the Myo7a+ cells are also PV+ in both the population of HCs regenerated from Lgr5+ progenitors and the population regenerated from Lgr5- SCs. **(E’,F’)** The hair bundle marker phalloidin in Lgr5+ progenitors and Lgr5- SCs. **(G,H)** The hair bundle marker espin1 in Lgr5+ progenitors and Lgr5- SCs. The yellow arrows show the long bundles and the white arrows show the short bundles. **(I,J)** The FM1-43 dye staining in Lgr5- SCs and Lgr5+ progenitors. Scale bars are 100 μm in **(A,B)**; 2 μm in **(C–F)**; and 5 μm in **(G–J)**.

FIGURE S2 **Lgr5+ progenitors have greater sphere-forming ability than Lgr5- SCs. (A,B)** The HC marker Myo6 labeled the newly regenerated HCs from the spheres formed by Lgr5- SCs and Lgr5+ progenitors. All of the Myo7a+ cells are also Myo6+ in both the population of HCs regenerated from Lgr5+ cells and the population regenerated from Lgr5- SCs. **(C,D)** The HC marker PV labeled the newly regenerated HCs from the spheres formed by Lgr5- SCs and Lgr5+ progenitors. All of the Myo7a+ cells are also PV+ in both the population of HCs regenerated from Lgr5+ progenitors and the population regenerated from Lgr5- SCs. **(A’,B’)** The hair bundle marker phalloidin in the spheres formed by Lgr5+ c progenitors and Lgr5- SCs. **(E,F)** The hair bundle marker espin1 in the spheres formed by Lgr5+ progenitors and Lgr5- SCs. The yellow arrows show the long bundles and the white arrows show the short bundles. **(G,H)** The FM1-43 dye staining in Lgr5- SCs and Lgr5+ progenitors. Scale bars are 20 μm in **(A,B)**; 5 μm in **(C–F)**; and 10 μm in **(G,H)**.
