# Supplementary figures and images for: Characterization of the Transcriptomes of Lgr5+ Hair Cell Progenitors and Lgr5- Supporting Cells in the Mouse Cochlea
Source: Front Mol Neurosci. 2017 Apr 26;10:122. doi: 10.3389/fnmol.2017.00122 (PMC5405134; doi:10.3389/fnmol.2017.00122)

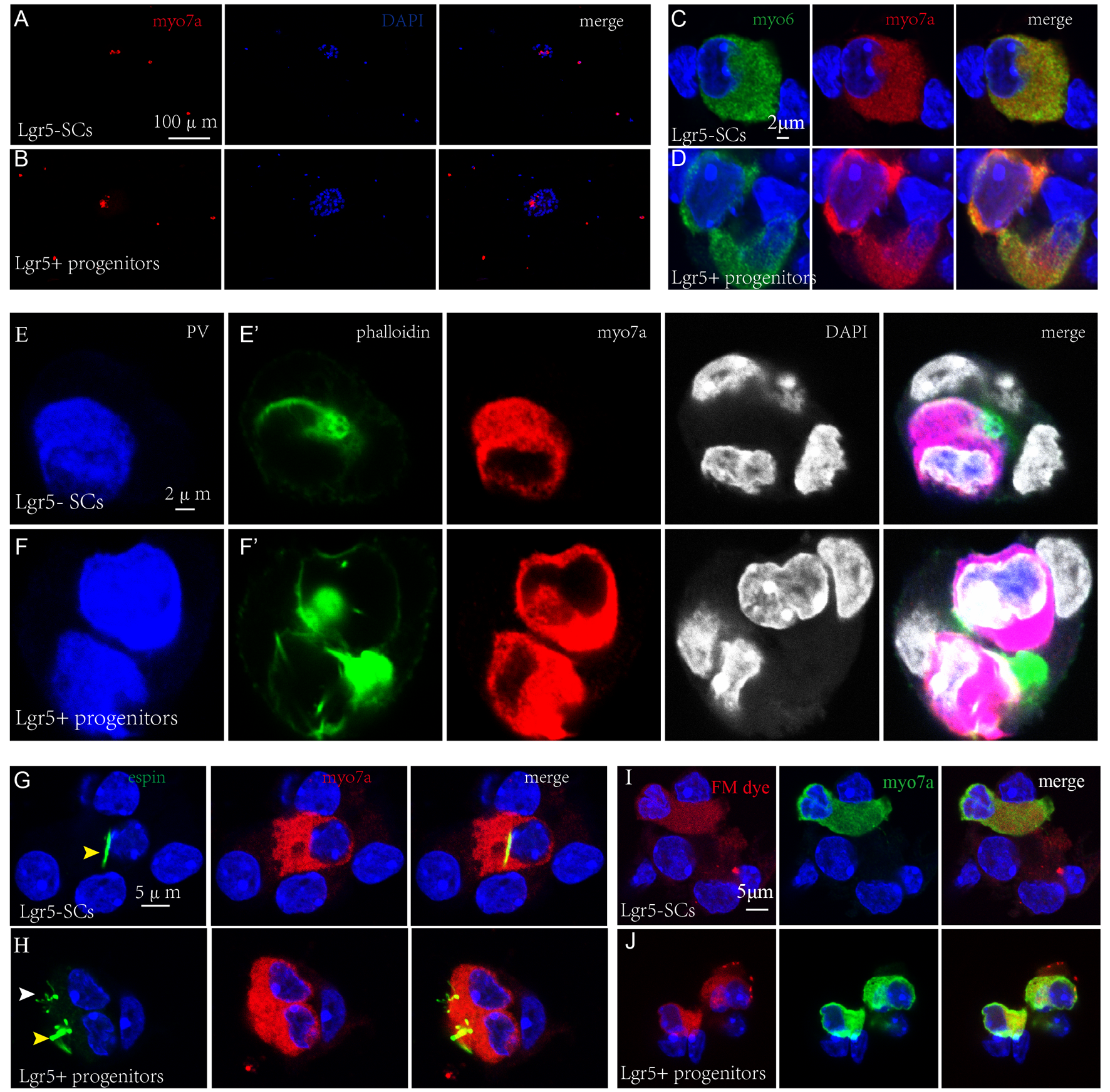

Supplement: FIGURE S1 — Lgr5+ HC progenitors generate more HCs compared to Lgr5- SCs in vitro. (A,B) After 10 days of culture, the low magnification images of the inside colonies and outside colonies showed that Lgr5+ progenitors generated more Myo7a+ colonies than Lgr5- SCs. (C,D) The HC marker Myo6 labeled the newly regenerated HCs in Lgr5- SCs and Lgr5+ progenitors. All the Myo7a+ cells are also Myo6+ in both the population of HCs regenerated from Lgr5+ cells and the population regenerated from Lgr5- SCs. (E,F) The HC marker PV also labeled the newly regenerated HCs in Lgr5- SCs and Lgr5+ progenitors. All of the Myo7a+ cells are also PV+ in both the population of HCs regenerated from Lgr5+ progenitors and the population regenerated from Lgr5- SCs. (E’,F’) The hair bundle marker phalloidin in Lgr5+ progenitors and Lgr5- SCs. (G,H) The hair bundle marker espin1 in Lgr5+ progenitors and Lgr5- SCs. The yellow arrows show the long bundles and the white arrows show the short bundles. (I,J) The FM1-43 dye staining in Lgr5- SCs and Lgr5+ progenitors. Scale bars are 100 μm in (A,B); 2 μm in (C–F); and 5 μm in (G–J). [file Image_1.TIF]

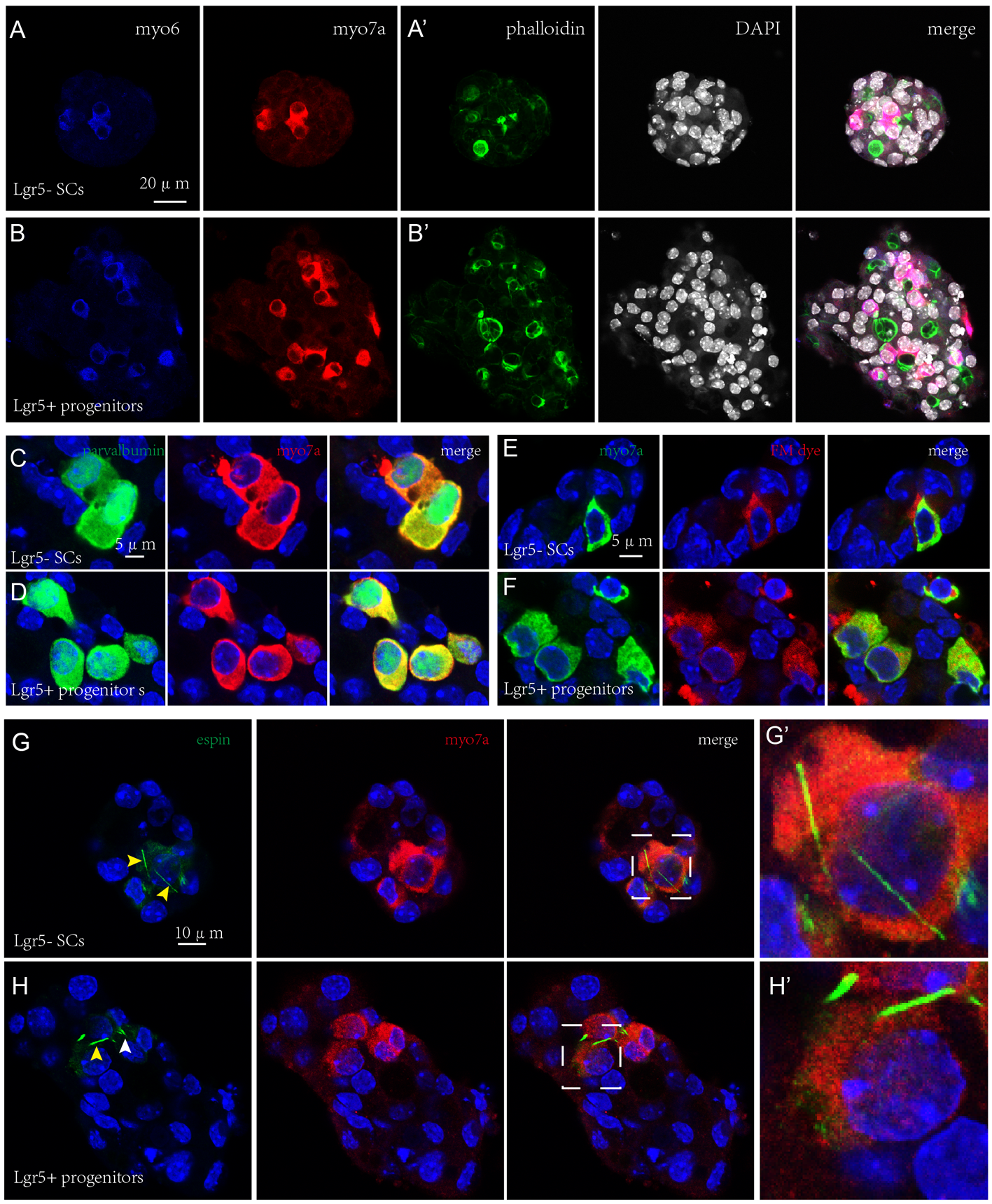

Supplement: FIGURE S2 — Lgr5+ progenitors have greater sphere-forming ability than Lgr5- SCs. (A,B) The HC marker Myo6 labeled the newly regenerated HCs from the spheres formed by Lgr5- SCs and Lgr5+ progenitors. All of the Myo7a+ cells are also Myo6+ in both the population of HCs regenerated from Lgr5+ cells and the population regenerated from Lgr5- SCs. (C,D) The HC marker PV labeled the newly regenerated HCs from the spheres formed by Lgr5- SCs and Lgr5+ progenitors. All of the Myo7a+ cells are also PV+ in both the population of HCs regenerated from Lgr5+ progenitors and the population regenerated from Lgr5- SCs. (A’,B’) The hair bundle marker phalloidin in the spheres formed by Lgr5+ c progenitors and Lgr5- SCs. (E,F) The hair bundle marker espin1 in the spheres formed by Lgr5+ progenitors and Lgr5- SCs. The yellow arrows show the long bundles and the white arrows show the short bundles. (G,H) The FM1-43 dye staining in Lgr5- SCs and Lgr5+ progenitors. Scale bars are 20 μm in (A,B); 5 μm in (C–F); and 10 μm in (G,H). [file Image_2.TIF]
